# Peroxisome Carrier SLC25A17: Potential Biomarker for Peroxisome Dysfunction and Human Disease

**DOI:** 10.3390/ijms27125448

**Published:** 2026-06-16

**Authors:** Arun Chhetri, Channy Park, Laxman Manandhar, Hyunsoo Kim, Raekil Park

**Affiliations:** Department of Biomedical Science and Engineering, Gwangju Institute of Science and Technology, Gwangju 61005, Republic of Korea; chhetri5@gm.gist.ac.kr (A.C.); channypark@gist.ac.kr (C.P.); laxmanandhar@gm.gist.ac.kr (L.M.); ruyhyunsookim@gm.gist.ac.kr (H.K.)

**Keywords:** SLC25A17, PMP34, PMP47, cofactor transport, peroxisome

## Abstract

Solute carrier family 25 (SLC25) is known to facilitate the transport of diverse metabolites across the mitochondrial and peroxisomal membranes. SLC25A17 is the only member of the SLC25 protein localized to peroxisomes; formerly known as PMP34, it also shares conserved sequence features with other SLC families. SLC25A17 was first described as an ATP transporter, but conflicting results regarding cofactor specificity in various experimental models obscure its precise function. Similarly, phenotypic differences between experimental models, such as mice and zebrafish, complicate the application of animal studies to humans. In particular, SLC25A17 deficiency is associated with peroxisomal dysfunction, and SLC25A17 expression is affected in various cancers and bipolar disorder, while the underlying molecular mechanisms remain unknown. Furthermore, it remains unclear whether altered SLC25A17 expression is a cause or consequence of human disease. This review provides an overview on current knowledge of SLC25A17, focusing on its known functions and emerging roles in human diseases. This may also help future studies in understanding its metabolic significance and disease pathogenesis.

## 1. Introduction

A total of fifty-three solute carrier family 25 (SLC25) proteins have been identified in humans, and they differ in the properties and sizes of substrates they transport [1,2,3]. SLC25 has been reported to facilitate the transport of amino acids, nucleotides, ions, protons, fatty acids, and carboxylates across the mitochondrial membrane through antiport, symport, and uniport activities [4,5,6,7,8]. While all other SLC25 proteins are located in the mitochondrial membrane, only solute carrier family 25 member 17 (SLC25A17) is localized to peroxisomes [9,10,11]. In mammals, it is also known as the peroxisomal membrane protein PMP34, and in yeast, its homolog PMP47 is present [12,13].

Peroxisomes are equipped with several sophisticated transport systems that enable them to carry out their metabolic functions [14,15,16,17]. One of the transport mechanisms in peroxisomes is carrier-mediated transport, which involves three ABC transporters (ABCD1, ABCD2, and ABCD3) and SLC25A17. These transporters allow the passage of bulky metabolites and essential cofactors across the peroxisomal membrane [10,17,18].

SLC25A17 was initially identified as an adenosine triphosphate (ATP) transporter; however, it is now recognized to facilitate the transport of various cofactors, including Coenzyme A (CoA), nicotinamide adenine dinucleotide (NAD^+^), NADP^+^, flavin adenine dinucleotide (FAD), flavin mononucleotide (FMN), ATP, heme, pyridoxal phosphate, and thiamine pyrophosphate [18,19], which are crucial for sustaining peroxisomal β-oxidation of very long-chain fatty acids (VLCFAs), pristanic acid, and bile acid intermediates [9,10,18]. Although SLC25A17 serves as a key transporter, other transporters, such as PXMP2 (a pore-forming protein) and PEX11, may also contribute to the passive diffusion of small metabolites (<400 Da) across the peroxisomal membrane [14,15]. Interestingly, emerging clinical data indicate that SLC25A17 is significantly upregulated in malignant tissues compared to corresponding normal counterparts [20,21]. Conversely, SLC25A17 deficiency is implicated in impaired peroxisomal function and lipid metabolism disorders [9,10]. Despite its clinical relevance, the contribution of SLC25A17 to disease progression and its specific role within peroxisomes remain to be fully elucidated. To address these gaps, this review delineates the structural characteristics, established functions, and disease associations of SLC25A17, and suggests its potential as a therapeutic target for future investigation.

## 2. Structure of SLC25A17

SLC25A17 was first described in 1998 by Wylin et al. as a homolog of *Candida boidinii* (*C. boidinii*) PMP47 based on sequence similarity [12], and was studied by Honsho and Fujiki as a model PMP to investigate topogenic signals [22].

Proteins of the SLC family share common sequence features [2,3]. According to the initial structural information for the ADP/ATP transporter of *Saccharomyces cerevisiae* (*S. cerevisiae*), the transporter is a monomeric, rod-shaped structure composed of three domains related by threefold pseudo-symmetry [23,24]. Each domain consists of an odd-numbered transmembrane α-helix, a loop containing short substrate-binding α-helices, and an even-numbered transmembrane (TM) α-helix, which together form a six-TM-helix barrel around a central transport pathway [23,24].

**Figure 1:**
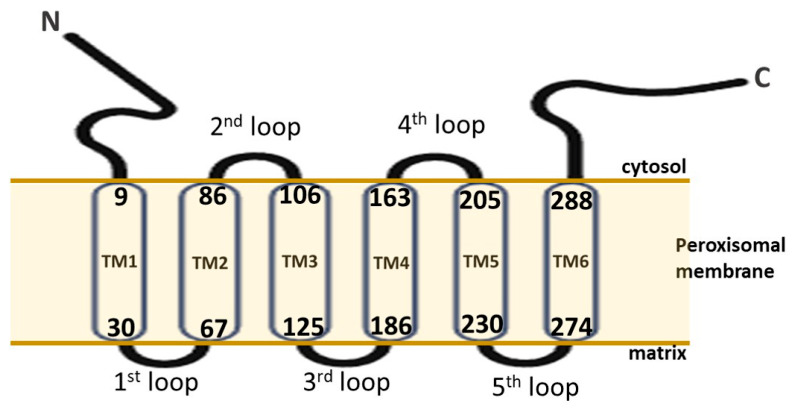
Topology map of SLC25A17. N-terminus “N” and C-terminus “C”.

The SLC25A17 gene is located on chromosome 22q13 [12], and the encoded protein has a molecular weight of 34 kDa and consists of 307 amino acids [12]. CHO-K1 cells transformed with FLAG-SLC25A17-HA suggest that both the N-terminus and C-terminus of SLC25A17 are oriented toward the cytosolic side of the peroxisomal membrane (Figure 1) [22].

SLC25A17 has six TM sequences from TM1 to TM6 (Figure 1). A fourth loop, consisting of amino acid sequences 164 through 204 located between TM4 and TM5, is essential for directing SLC25A17 into the peroxisome [22]. However, this loop alone is not sufficient for proper insertion into the membrane. For SLC25A17 to be accurately targeted and integrated into the peroxisomal membrane, one of the TM1-TM3 or TM4-TM6 sequences is required along with the fourth loop [22].

**Figure 2:**
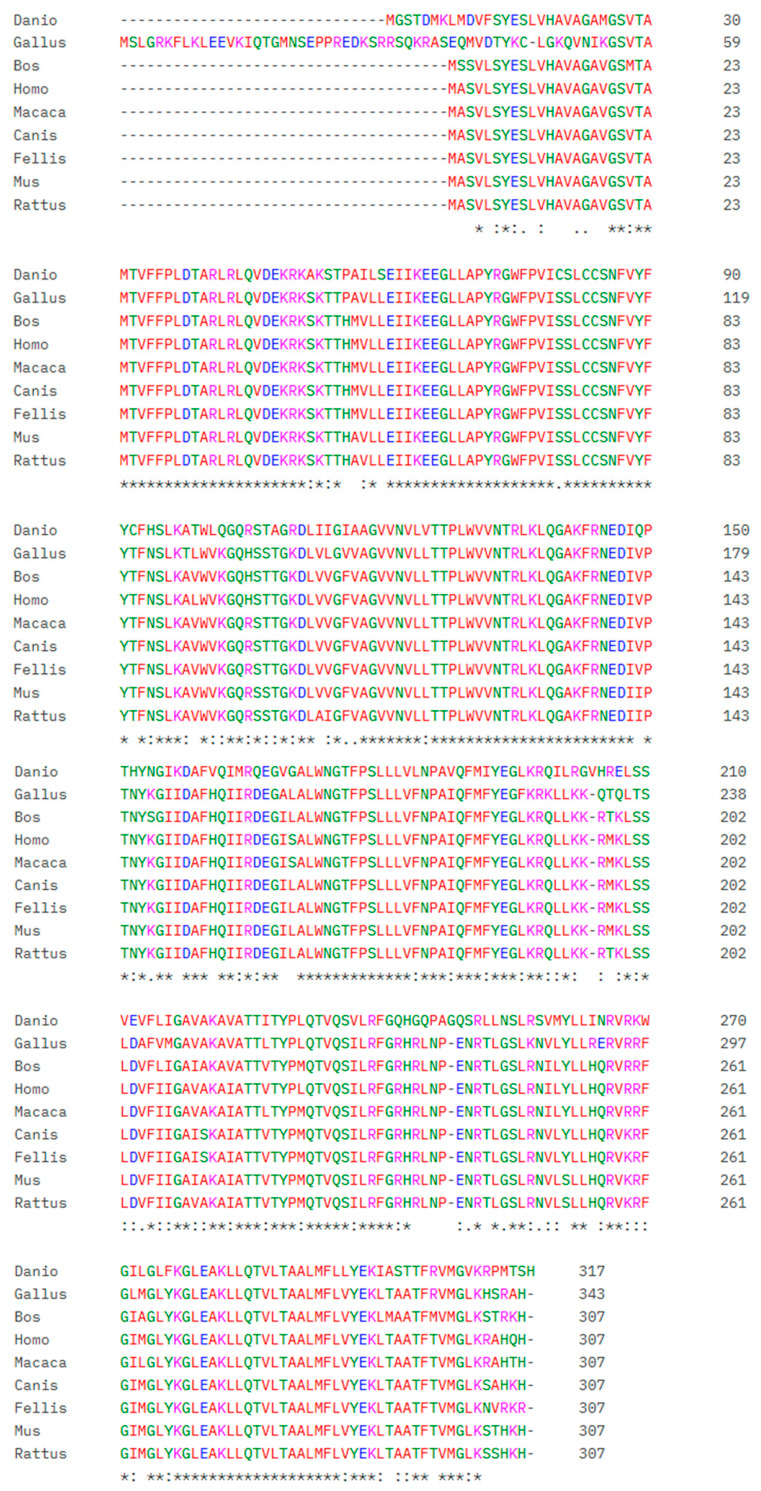
Comparison of the sequence homology of SLC25A17 across nine species using Clustal Omega multiple sequence alignment software (v.1.2.4). The accession numbers of the SLC25A17 species are as follows: NP_006349.1, NP_035529.1, NP_001092731.2, NP_001119741.1, NP_001244445.1, NP_001039413.1, XP_038535691.1, XP_040525033.1, and XP_003989333.1. “*” Fully conserved residue, “:” conservation between groups of strongly similar properties, “.” conservation between groups of weakly similar properties, “ ” position with residues that have different properties.

SLC25A17 exhibits a high degree of sequence homology among *Homo sapiens*, *Mus musculus*, *Danio rerio*, *Rattus norvegicus*, *Macaca mulatta*, *Bos taurus*, *Canis lupus familiaris*, *Gallus gallus*, and *Felis catus*, indicating that it is highly conserved across diverse evolutionary species (Figure 2) [25].

## 3. Functions of SLC25A17

Recombinant purified SLC25A17 reconstituted into a liposome has been used to determine its transport properties [18,19]. In addition, SLC25A17 function has been studied in physiological contexts using animal models, including mice and zebrafish [9,10]; however, a consensus regarding its definitive substrate specificity and function has yet to be reached. Therefore, in this section, we also propose potential hypotheses to reconcile the discrepant phenotypes observed across different SLC25A17-deficient animal models.

### 3.1. Transport

#### 3.1.1. Cofactor Transporter

NAD^+^ and FAD are essential cofactors for cellular respiration and act as electron carriers in ATP production, while CoA is an essential acyl-group carrier. Peroxisomes import these cofactors via specific protein transporters such as SLC25A17 [10,18].

Recombinant SLC25A17 purified from HepG2 was reconstituted into a liposome and tested for its potential substrate [18]. The results show that SLC25A17 can transport AMP, ADP, CoA, NAD^+^, FAD and FMN via a counter-exchange mechanism [18]. The homo-exchange of [^14^C]AMP/AMP and [^14^C]ADP/ADP was observed when the same substrate was present inside and outside the liposome reconstituted with SLC25A17. The uptake of [^14^C]AMP into these liposomes was highest when the internal substrates were AMP, CoA, and adenosine 3′, 5′ diphosphate (PAP) [18]. Similarly, fluorescent FAD and FMN were taken up into liposomes in exchange for internal AMP, FAD, CoA, and PAP. [^3^H]NAD^+^ and [^14^C]ADP were also transported into liposomes containing internal AMP, FAD, FMN, CoA, and PAP. It has been suggested that in the absence of external substrates such as CoA or AMP, AMP and FAD efflux from liposomes is significantly reduced [18].

CoA transport by SLC25A17 was confirmed in studies using zebrafish. Liposomes reconstituted with zebrafish-derived SLC25A17 were able to transport CoA [10], and the swim bladder of zebrafish, which did not inflate by SLC25A17 knockdown, was rescued to the normal state upon CoA injection [10]. However, the NAD^+^ transport capacity of SLC25A17 is still unclear. In contrast to liposomes reconstituted with SLC25A17 from HepG2 cells, those reconstituted with SLC25A17 from zebrafish did not transport NAD^+^ [10]. Furthermore, in vivo injection of NAD^+^ did not restore the defective swim bladder of SLC25A17 knockdown zebrafish [10]. These conflicting study results might arise from the different experimental methods used to examine SLC25A17 function. The in vitro liposome reconstitution assay utilizes purified human proteins embedded within artificial membranes to evaluate substrate transport under strictly controlled conditions. In contrast, zebrafish studies are based on the complex in vivo system where substrate transport kinetics can be modulated by diverse physiological factors within the living organism. Nevertheless, a comparative synthesis of these findings suggests that while SLC25A17 is capable of transporting various substrates, CoA serves as the preferred substrate within a physiological and biological context. Although SLC25A17 is a peroxisomal cofactor transporter, its exact substrate specificity continues to elicit conflicting views among investigators. Given that no alternative NAD^+^ transporters have been identified in mammalian peroxisomes to date, fully uncovering the physiological role of SLC25A17 is becoming increasingly difficult.

#### 3.1.2. ATP Transporter

Peroxisomes lack the machinery to produce ATP and depend on import from cytosol, and ATP within peroxisomes is essential for peroxisomal β-oxidation of fatty acid [26]. SLC25A17 functions as an ATP/ADP (or AMP) exchanger [19]. Liposome reconstitution assay showed that purified human SLC25A17 can uptake ATP in the presence of AMP and ADP as a counter-exchange ion [19]. However, similar studies have reported that ATP transport by SLC25A17 is negligible compared to cofactor transport [18]. This discrepancy in substrate preference is likely attributable to differences in the specific experimental conditions and the structural integrity of the SLC25A17 proteins utilized across these independent studies [18,19].

#### 3.1.3. Glutathione Redox Couple

The NADPH/NADP^+^ is essential for the glutathione (GSH) redox cycle, as GSH reductase uses NADPH to regenerate reduced GSH from its oxidized form (GSH disulfide, GSSG). GSH scavenges free radicals and other reactive oxygen species (ROS) [27], and it is synthesized in cytosol by γ-glutamylcysteine synthase and GSH synthetase and is subsequently distributed to various subcellular organelles. In mitochondria, SLC25A39 and SLC25A40 function as GSH transporters [28]. Although loss of PXMP2 and PEX11B reduces the peroxisomal GSH level, no specific GSH transporters have yet been identified in the mammalian peroxisomal membrane [29]. Both SLC25A17-deficient HeLa cells and T antigen-transformed mouse embryonic fibroblasts revealed a significant reduction in the peroxisomal GSSG/GSH redox state without altering the mitochondrial redox state [30]. Moreover, reintroducing SLC25A17 or PMP47 expression in SLC25A17-deficient cells restored the peroxisomal GSSG/GSH redox state disturbances, suggesting that the two proteins, SLC25A17 and PMP47, share orthologous functions in regulating peroxisomal GSH redox metabolism [30]. However, the authors of this study do not demonstrate whether SLC25A17 acts as a peroxisomal GSH transporter.

### 3.2. Regulating Peroxisomal Function

#### 3.2.1. Oxidation of Branched-Chain Fatty Acid (BCFA)

BCFAs, such as phytanic acid, cannot be directly oxidized by β-oxidation, because a methyl group is present on the third carbon atom [31]. The first step of BCFA oxidation is removing the alpha-carbon through alpha-oxidation, which occurs only in peroxisomes [9,31]. Phytanic acid is converted to pristanic acid through a series of enzymatic reactions. The pristanoyl-CoA generated from pristanic acid is converted within peroxisomes into propionyl-CoA, acetyl-CoA, and 4,8-dimethylnonanoyl-CoA, which are then transported to the mitochondria for further oxidation [31].

SLC25A17 knockout (KO) mice did not show significant changes in peroxisome function and phenotype compared to control mice [9]. However, when phytol was fed to examine BCFA oxidation, hepatic inflammation and microvesicular steatosis occurred [9]. In these SLC25A17 KO mice, phytol intake led to the accumulation of phytanic acid and pristanic acid, as well as phytanoyl-CoA and pristanoyl-CoA, in the liver [9]. This suggests that SLC25A17 is essential for breaking down phytol-derived BCFAs. However, SLC25A17-deficient mice did not show changes in the peroxisomal level of CoA, ATP, and NAD^+^ [9]. Although the authors of this study could not clearly determine whether the phenotype observed in SLC25A17 KO mice was due to a defect in enzyme function or in cofactor import, they suggested that sterol transport protein-X (SCP-X) activity may be reduced in the absence of CoA import [9]. This may have led to the accumulation of BCFA intermediates and inhibited the oxidation of phytanic acid and pristanic acid.

These findings highlight the importance of SLC25A17 in BCFA metabolism and suggest a potential link to human peroxisomal disorders, such as Refsum syndrome and Zellweger syndrome, which are characterized by the accumulation of phytanic acid and pristanic acid in the tissues [31].

#### 3.2.2. Oxidation of VLCFA

VLCFA oxidation with 20 or more carbon atoms occurs primarily in peroxisomes [32,33]. ABCD1 transports VLCFA-CoA into peroxisomes, while SLC25A17 ensures that the CoA pool within the peroxisomes is available for subsequent oxidation cycles [10,34].

A study in zebrafish revealed that SLC25A17 knockdown increases the amount of VLCFAs, such as C24/C22 and C26/C22, at 3 and 5 days post fertilization (dpf) in zebrafish embryos [10]. In SLC25A17-deficient zebrafish embryos, the expression of acyl-CoA oxidase 1, D-functional protein, sterol carrier protein 2, and alkylglycerone phosphate synthase is reduced, accompanied by lipid accumulation [10]. By contrast, VLCFA metabolism was not affected in SLC25A17-deficient mice [9]. Similarly, an in vitro experiment showed that the oxidation rate of hexacosane (C26:0) in SLC25A17-deficient HEK-293 and HeLa cells was not affected compared to control cells [30], which suggests a distinct role of SLC25A17 in VLCFA metabolism across experimental models.

Mechanistically, SLC25A17 deficiency likely impairs the availability of essential cofactors required for the optimal function of VLCFA-oxidizing enzymes within the peroxisomal matrix. The initial step of peroxisomal beta-oxidation converts VLCFA-CoA to enoyl-CoA by ACOX1, a process strictly requiring FAD as a cofactor [35]. Subsequently, D-bifunctional protein catalyzes a second dehydrogenation reaction that requires NAD^+^ [35], followed by a thiolysis reaction mediated by SCP-X, which requires free CoA. Notably, all of these cofactors are established substrates transported by human SLC25A17 [18]. The absence of a VLCFA phenotype in mice and human cells, despite the loss of SLC25A17, strongly implies the existence of robust, mammalian-specific compensatory mechanisms. In mammalian systems, alternative passive or active transport pathways—such as the channel-forming peroxisomal membrane protein PXMP2 or other uncharacterized carriers—might sufficiently maintain basal peroxisomal cofactor pools under resting conditions. Additionally, human peroxisomal proteins intimately influence VLCFA homeostasis; for instance, defects in any of the 14 *PEX* genes involved in mammalian peroxisomal assembly impair organelle biogenesis, resulting in systemic VLCFA accumulation [36]. Future investigations are warranted to explore the role of SLC25A17-mediated transport in peroxisomal VLCFA metabolism and determine whether it contributes to human VLCFA-disorders.

#### 3.2.3. Oxidation of Medium-Chain Fatty Acid (MCFA)

In higher organisms, MCFA oxidation involves the β-oxidation process occurring in mitochondria, during which MCFAs are broken down into acetyl-CoA for energy production. Therefore, SLC25A17 knockdown does not attenuate MCFA oxidation in mice or zebrafish [9,10].

Peroxisomal adenine nucleotide transporter 1 (ANT1) is known to be present in *S. cerevisiae*. Liposome reconstitution assay revealed that ANT1 is an ATP transporter in yeast [37], and deleting ANT1 in *S. cerevisiae* reduces β-oxidation of MCFAs [19]. Similarly, PMP47-deficient *C. boidinii* cannot grow using MCFAs, such as laurate (C12:0) [38]. This MCFA defect in *S. cerevisiae* is partially restored (approximately 60%) through human SLC25A17 overexpression [19]. Furthermore, expressing *C. boidinii* PMP47, a homolog of human SLC25A17, in ANT1-deficient *S. cerevisiae* can restore the ability to utilize MCFAs [37]. This indicates that SLC25A17 and PMP47 act on MCFA metabolism by providing ATP.

In yeast, FAO occurs only in peroxisomes [39]. Acetyl-CoA produced in peroxisomes is transported to the mitochondria or cytoplasm for biological processes. MCFAs can be transported directly into the yeast peroxisomes by passive diffusion and, once inside, should be activated by fatty acyl-CoA synthetase (FAA2) before undergoing further oxidation [40]. The conversion of MCFAs to MCFA-CoA by FAA2 requires ATP and CoA within the peroxisome, which is likely provided by PMP47 within the yeast peroxisome [37,38]. Taken together, the difference in MCFA oxidation between PMP47-deficient *C. boidinii* and SLC25A17-deficient mice or zebrafish lies in the subcellular organelle responsible for metabolism.

The physiological relevance of impaired laurate β-oxidation in PMP47-deficient *C. boidinii* could be demonstrated with studies on acyl-CoA synthetase medium-chain family member 3 (ACSM3) KO mice [41]. These mice show that hepatic lauric acid accumulation induces metabolic syndrome through the lauric acid–HNF4a–p38 MAPK axis, suggesting that altered SLC25A17 function may produce similar metabolic effects and necessitate future investigation [41].

On the other hand, long-chain fatty acids (LCFAs) are activated in the cytoplasm by fatty-acyl-CoA synthases (FAA1 and FAA4) in yeast, which are then transported in the form of LCFA-CoA to the peroxisomes via ABC transporters (PXA1 and PXA2) for further oxidation [40]. Therefore, PMP47 deficiency does not affect LCFA oxidation in *C. boidinii*.

#### 3.2.4. Effect on Peroxisome Number

Peroxisome number is regulated through biogenesis and degradation processes, and there are some mechanistic differences between yeast and higher eukaryotes [42,43,44,45,46,47,48]. Loss of PMP47 reduces the number of peroxisomes in *C. boidinii* cultured on several media, including those containing oleate, palmitate, or methanol/glycerol, suggesting that PMP47 is essential for peroxisomal abundance in *C. boidinii* [13,38]. By contrast, studies in SLC25A17-deficient zebrafish and mice have shown no effect on peroxisome number [9,10].

In yeast, the pre-peroxisomal vesicles involved in the de novo biogenesis of peroxisome come exclusively from the endoplasmic reticulum (ER) [45]. Since yeast lacks mitochondria-derived pre-peroxisomal vesicles, this limitation may have led to a decrease in peroxisome number in PMP47-deficient *C. boidinii*. However, the additional biogenesis from mitochondria is an extra process of peroxisome quality control present in higher eukaryotes but absent in yeast [45,49,50,51]. Mitochondria-derived vesicles can deliver certain PMPs to pre-peroxisomal vesicles in mammalian cells and play a significant role in mammalian peroxisome biogenesis [45,49,50,51]. This alternate route of PMP trafficking may help maintain peroxisome number in SLC25A17-deficient zebrafish and mice.

Furthermore, selective autophagy in peroxisomes, also known as pexophagy, is a process which removes damaged peroxisomes and maintains peroxisome homeostasis. The proper localization of peroxisome protein is necessary for sustaining peroxisome integrity [42]. PMP47-deficient *C. boidinii* loses the ability of dihydroxyacetone synthase protein to properly fold and localize within the peroxisome [13]. In this case, it can be proposed that proper localization of other PMPs is also disrupted, which may lead to increased pexophagy and peroxisome reduction [13,38].

Moreover, in yeast, phosphorylation at specific residues of Atg30 and Atg36 bound to peroxisome acts as the molecular tag for pexophagy [46,47,48]. In mammals, PEX5 and PMP70 ubiquitination acts as a signal for selective autophagy [46,52]. However, the direct influence of PMP47 and SLC25A17 on the regulation of pexophagy-related proteins has not yet been studied. It can be speculated that these proteins are differentially activated in PMP47-deficient *C. boidinii*, which may have led to the decrease in the number of peroxisomes compared to SLC25A17-deficient mice or zebrafish. Therefore, these results suggest that SLC25A17 and PMP47 play distinct roles in regulating peroxisome number.

### 3.3. Others

Peroxisome disorders cause developmental abnormalities [53]. Zebrafish have two SLC25A17 genes, SLC25A17 and SLC25A17-like (SLC25A17L), which are highly conserved (~80%) compared to human SLC25A17 [10]. Knockdown of either SLC25A17 or SLC25A17L results in a similar embryonic phenotype. These two genes are redundant and perform identical functions, and this redundancy may explain why loss of SLC25A17 can produce a stronger phenotype in zebrafish than in mice. During zebrafish embryonic development, the overexpression of one gene compensates for the deficiency of the other gene [10]. Knockdown of SLC25A17 leads to reduced body length, impaired myelination of peripheral nerves in the head and trunk, failure of swim bladder inflation, and the downregulation of genes involved in the formation and growth of endoderm-derived organs [10].

SOX2 is an early marker gene for epithelial and endodermal organ development [10,54], and its expression is dependent on p300 acetylase activity [55], which in turn depends on the presence of acetyl-CoA [56]. Similarly to how impaired FAO in ACOX1 KO mice restricts cytosolic acetyl-CoA availability and consequently impairs acetylation in raptors [57], impaired peroxisomal FAO in SLC25A17 may decrease acetyl-CoA pools, thereby impairing SOX2 acetylation and expression in SLC25A17 KO zebrafish [55]. Thus, SLC25A17 could indirectly influence embryogenesis and organ development in zebrafish by affecting the peroxisomal fatty acid oxidation and acetyl-CoA pools required for transcriptional regulation of genes. In addition, it is accompanied by reduced plasmalogen synthesis, which is essential for neural growth and function [10].

### 3.4. Possible Hypotheses for Different Phenotypes in SLC25A17-Deficient Animal Models

As described above, SLC25A17 knockdown in zebrafish induces FAO defects, organ malformation, and lipid accumulation, which are reversed by CoA injection [10]. By contrast, SLC25A17-deficient mice, without being fed phytol, do not exhibit these phenotypes and, unexpectedly, show no changes in peroxisome cofactors [9]. It is presumed that several compensatory mechanisms maintain cofactor homeostasis and peroxisome FAO in SLC25A17 KO mice. One possibility is the import of enzymes together with their cofactors. Peroxisomal proteins can undergo folding in the cytoplasm, acquire cofactors, form oligomers, and then enter peroxisomes. Upon entering peroxisomes, these proteins bring the bound cofactors along with them [58,59]. A similar mechanism could help maintain the cofactor pool within peroxisomes in SLC25A17 KO mice.

Alternate mechanisms of generating cofactors, such as the peroxisomal malate-oxaloacetate shuttle system and acyl-CoA thioesterases capable of generating NAD^+^ and CoA, respectively, may function differently in the peroxisomes of SLC25A17KO mice but not in zebrafish, which contribute to maintaining peroxisomal cofactor levels and phenotypic differences between the two organisms [60,61].

Furthermore, the dual targeting of mitochondrial cofactor transport proteins through gene duplication, alternative transcription, or post-translational modification may enable their localization to peroxisomes, which can maintain cofactor homeostasis and support FAO in SLC25A17 KO mice [62,63]. Similarly, sequence comparisons of mouse SLC25A17 (NP_035529.1) and zebrafish (NP_001430392.1 and NP_001092731.2) using NCBI Protein Blast showed homologies of 66.67% and 74.34%, respectively. Therefore, while core functions were conserved in both organisms, it is possible that there were some changes in the cofactor transport property.

The phenotypic differences between two experimental models may also be attributed to methodological differences employed to disrupt SLC25A17 function. In zebrafish, SLC25A17 knockdown was achieved using morpholino antisense oligonucleotides, which temporarily block RNA translation or alter pre-mRNA splicing. In contrast, the mice gene-trap knockout produces a stable loss-of-function allele. Studies have reported that morpholino-induced phenotypes are not necessarily recapitulated in genetic mutants [64], leading to recommendation that morpholino-derived phenotypes be validated through comparison with genetic mutant whenever possible [65].

In addition, species-specific differences in the peroxisomal metabolism may contribute to distinct phenotypes. Mammalian peroxisomes contain enzymes such as BAAT (bile acid-CoA: amino acid N-acyltransferase) and ZADH2/PTGR3 (zinc-dependent alcohol dehydrogenase 2/prostaglandin reductase 3), whereas zebrafish possess peroxisomal malate synthase (Mls1) that utilizes CoA as a substrate [66]. This unique metabolic feature may increase the dependence of zebrafish peroxisome on SLC25A17-mediated CoA transport, potentially making zebrafish more susceptible to SLC25A17 deficiency than mammals. However, these mechanisms remain to be elucidated in future studies.

## 4. SLC25A17-Related Human Diseases

**Figure 3:**
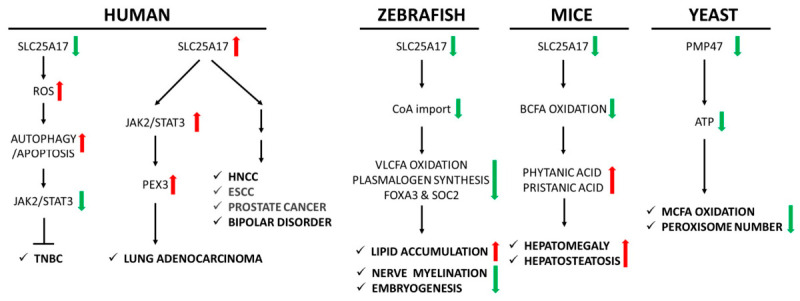
Defects related to SLC25A17.

SLC25A17 deficiency is linked with a loss of peroxisome function, but not with a decrease in the number of peroxisomes [9,10]. SLC25A17 is significantly upregulated in tumor tissue compared with normal tissue in certain cancers, including triple-negative breast cancer (TNBC), esophageal squamous cell carcinoma (ESCC), head and neck squamous cell carcinoma (HNSCC) and enzalutamide-resistant prostate cancer cells [20,67,68,69]. Additionally, increased SLC25A17 expression is correlated with poor overall survival in these cancers. Most cancer studies using RNA-seq analysis have shown changes in SLC25A17 transcript levels across various cancers [20,67,69]. However, since transcript levels alone cannot accurately predict protein abundance and transport function, uncertainty remains regarding the role of SLC25A17 in disease progression, and suggests the need for further research [67,69,70,71,72,73]. Studies employing SLC25A17 knockdown and overexpression could provide more detailed mechanistic insights in cancer models. Nevertheless, SLC25A17 appears to orchestrate context-dependent effects on cancer dynamics by influencing cellular ROS homeostasis, lineage differentiation, and peroxisomal lipid metabolism [21,68,70]. In this section, we introduce SLC25A17-related disorders, including several cancers, human papillomavirus infection, circadian rhythms, and bipolar disorder (Figure 3).

### 4.1. Triple-Negative Breast Cancer (TNBC)

Evidence showing an association between SLC25A17 and human TNBC is based on public datasets, TCGA, and GEO [68]. Currently, information regarding TNBC and SLC25A17 is based only on in vitro experiments and mouse experiments. Knockdown of SLC25A17 inhibits TNBC in vitro, and similarly, in a mouse model injected sub-cutaneously with the human TNBC cell line MDA-MB-231, the group with inhibited SLC25A17 expression showed a reduction in both tumor volume and weight [68].

Mechanistically, SLC25A17 is known to contribute to the maintenance of peroxisomal redox homeostasis. In TNBC, SLC25A17 knockdown increases ROS, which inhibit the JAK2/STAT3 pathway and suppress cancer cell proliferation [68]. Knockdown of SLC25A17 in breast cancer cells increases autophagosome formation and increases the expression of autophagy-related proteins LC3 and Beclin-1, thereby inducing autophagy. Likewise, the expression of apoptosis-related proteins BAX, cleaved caspase-3, and cleaved PARP is increased [68]. Treatment with ROS inhibitor N-acetyl-L-cysteine restores JAK2/STAT3 pathway activation and reduces autophagy and apoptosis in SLC25A17 knockdown breast cancer cells [68]. Similarly, increased SLC25A17 expression is associated with activation of the JAK2/STAT3 pathway and progression of lung adenocarcinoma [74]. Therefore, these findings suggest that SLC25A17 is not merely a transcription marker but an active driver of cancer progression, and could potentially be a therapeutic target for cancer treatment.

### 4.2. Neuroblastoma

Neuroblastoma (NB) is a rare cancer that arises from nerve tissue, primarily affecting children under the age of five. In contrast to other cancers described in this section, microarray analysis from multiple neuroblastoma cohorts have shown that the decreased expression of the SLC25A17 gene is directly correlated with poor overall survival and relapse-free survival rates in children with neuroblastoma [70]. Similarly, transcriptional analysis of metastatic site-derived aggressive cells showed decreased SLC25A17 expression [70]. Such cancer-type-specific roles of SLC25A17 likely reflect the discrepancies in their cellular origin. Neuroblastoma arises from neural crest-derived cells and is functionally characterized by differentiation arrest [75]. In contrast, TNBC, ESCC, and HNSCC are epithelial malignancies that progress primarily through uncontrolled proliferation. Peroxisome abundance significantly increases during differentiation of human-derived neuroblastoma SH-SY5Y cells into neuron-like cells, suggesting the crucial role of peroxisomal metabolism in this lineage commitment, although its precise underlying mechanism remains unknown [76]. Therefore, the disruption of peroxisomal homeostasis under conditions of reduced SLC25A17 expression may impair normal cellular differentiation and consequently contribute to neuroblastoma pathogenesis. Taken together, these insights suggest that SLC25A17 may function as a context- and differentiation-dependent tumor suppressor specifically in neuroblastoma. Interestingly, progesterone-induced decidual protein (DEPP) is localized to peroxisomes in neuroblastoma cells, where it increases ROS and sensitizes tumors to apoptosis [77]. This mechanism is similar to ROS-mediated apoptotic cell death in SLC25A17 knockdown TNBC.

### 4.3. Esophageal Squamous Cell Carcinoma (ESCC)

Peroxisomes play a complex role in ESCC. For example, certain drugs targeting the peroxisomal pathway, such as tegaserod maleate, can inhibit ESCC growth by inhibiting peroxisome function [78]. Proto-oncogene pituitary tumor-transforming gene (PTTG1) increases in many different cancers including breast cancer, pituitary adenomas, and thyroid tumorigenesis [79,80,81], and it is taken as a biomarker for these cancers. Multiple datasets, including gene microarray, public RNA-seq, and in-house RNA-seq on ESCC identified that PTTG1 expression shows a strong correlation with SLC25A17 in ESCC [69]. Considering these points, SLC25A17 may be a biomarker for ESCC, and further in-depth studies are needed in the future.

### 4.4. Head and Neck Squamous Cell Carcinoma (HNSCC)

Pan-cancer analysis of SLC25A17 expression revealed that it was approximately 2-fold higher in HNSCC tumor tissues compared to normal tissues [67]. In single-cell RNA sequencing analysis of HNSCC, infiltration of memory resting CD4^+^ T cells and M1 macrophages was positively correlated with SLC25A17 expression, whereas infiltration of CD8^+^ T cells, regulatory T cells, and neutrophils was negatively correlated [67]. T-distributed stochastic neighbor embedding (t-SNE) analysis from HNSCC shows that SLC25A17 is expressed in malignant tumor cells, immune cells, and stromal cells, suggesting that SLC25A17 could serve as a potential biomarker linked to the tumor microenvironment in HNSCC [67]. Patients with high SLC25A17 expression were found to be more sensitive to certain anticancer chemotherapeutic agents, such as cisplatin, docetaxel, gemcitabine, and paclitaxel, which target DNA and microtubules [67].

### 4.5. Prostate Cancer

In prostate cancer, evidence supporting the association with SLC25A17 was identified through the analysis of RNA sequencing from The Cancer Genome Atlas (TCGA) and the Genotype-Tissue Expression (GTEx) project and patient survival data [21]. SLC25A17 knockdown has been shown to suppress the proliferation and migration of enzalutamide-resistant cells, induce G1/S phase cell cycle arrest, and promote apoptosis via BAX upregulation [21]. Furthermore, SLC25A17 inhibition reduces the expression of fatty acid synthase (FASN) and acetyl-CoA carboxylase (ACC), which consequently lowers the levels of lactic acid, triglycerides, and citric acid within these resistant cells. Since proliferating cancer cells depend on increased lipid synthesis for cell membrane production, these findings indicates that SLC25A17 supports fatty acid metabolism to sustain survival and confer drug resistance in enzalutamide-resistant prostate cancer cells [21]. In addition, gene network analysis showed that SLC25A17 interacts with androgen receptor, tumor protein 53, tumor protein 63, SWI/SNF-related BAF Chromatin Remodeling Complex Subunit ATPase 4, E74-like ETS transcription Factor 1, E2F transcription factor 1, Nanog homeobox, and SRY-box transcription factor 17 [20,21]. Therefore, SLC25A17 has emerged as a potential target to improve cancer treatment because of its involvement in drug resistance, such as enzalutamide resistance in prostate cancer, and tumor development.

### 4.6. Human Papillomavirus (HPV) Infection

Approximately 5% of human cancers are caused by HPV infection. It was confirmed that SLC25A17 plays a key role in HPV infection through CRISPR-Cas9 screening using the sgRNA library [72]. In this method, cells are transfected with the sgRNA library for gene suppression, transfected with HPV-PsV-GFP-dTK for HPV infection, and finally treated with ganciclovir to select viable cells that are not virus-infected. Surviving cells showed abundant expression of sgSLC25A17, suggesting that SLC25A17 can be a potential gene involved in viral invasion. This was further verified through KO experiments using two independent gRNAs targeting SLC25A17. SLC25A17 KO significantly reduced HPV infection in both 293FT cells and HeLa cells [72]. However, future studies may determine whether SLC25A17 acts as a direct receptor for HPV infection or is the effect of dysregulated peroxisomal fatty acid metabolism and cofactors.

### 4.7. Circadian Rhythms

Circadian rhythms (the body’s internal clock) and peroxisomes are closely linked, and this clock regulates peroxisomal matrix protein import in glial cells. The inability of peroxisomal matrix protein import in cortex glial cells causes lipid accumulation and sleep disorder [82]. A 2018 genome-wide association study of more than 70,000 UK Biobank participants found an association between specific genetic variants within the SLC25A17 gene region and lower relative amplitude (RA) of the rest-activity cycle. These regions contain three SNPs that correspond to two gene loci, namely neurofascin on chromosome 1 and SLC25A17 on chromosome 22 [71].

### 4.8. Bipolar Disorder (BD)

Bipolar disorder is not directly caused by a genetic defect in peroxisomes. However, it is associated with reduced synthesis of essential fatty acids, such as docosahexaenoic acid (DHA), which are essential for brain health [83]. A decrease in DHA was also reported in SLC25A17 KO mice fed phytol [9]. Furthermore, the SLC25A17 gene has been identified as a genetic locus associated with an increased risk of bipolar disorder (BD), particularly in both bipolar I and II subtypes. This association was revealed through genome-wide association studies (GWASs) and integrated genome-wide analysis [73]. Therefore, further study is needed to determine whether single-nucleotide polymorphism at the SLC25A17 locus is associated with DHA reduction and contributes to BD development.

## 5. Conclusions

Despite significant progress in identifying and characterizing SLC25 transporters, a more detailed and rigorous functional characterization of SLC25A17 remains critically necessary. Such targeted research is vital to reconcile conflicting, model-dependent experimental outcomes and to confirm the existence of alternative or redundant peroxisomal transport proteins. Currently, oncological and clinical investigations of SLC25A17 are in their infancy, leaving uncertainties regarding its downstream molecular mechanisms. Therefore, further robust mechanistic studies are required to elucidate these pathways and to rigorously evaluate the clinical utility of SLC25A17 as both a diagnostic biomarker and a therapeutic avenue. By addressing these knowledge gaps, future research may successfully clarify the exact roles of SLC25A17 and its relevance to human diseases.

## Data Availability

No new data were created or analyzed in this study. Data sharing is not applicable to this article.

## Abbreviations

The following abbreviations are used in this manuscript:

SLC25A17	solute carrier family 25 member 17
PMP34	peroxisomal membrane protein 34
PMP47	peroxisomal membrane protein 47
PEX	peroxin
ABCD	ATP binding cassette subfamily D
MCFA	medium-chain fatty acid
BCFA	branched-chain fatty acid
VLCFA	very-long-chain fatty acid
HNSCC	head and neck squamous cell carcinoma
ESCC	esophageal squamous cell carcinoma
TNBC	triple-negative breast cancer
